# The detection of *Edwardsiella tarda* by aptamer-based qPCR

**DOI:** 10.3389/fvets.2025.1635525

**Published:** 2025-07-23

**Authors:** Yue Bai, Xuefei Li, Wei Yan, Lingmin Zhao, Lixing Huang, Qingpi Yan, Jiaen Wang, Qibiao Weng, Jiang Zheng

**Affiliations:** ^1^State Key Laboratory of Mariculture Breeding, Engineering Research Centre of the Modern Technology for Eel Industry, Ministry of Education, Fisheries College of Jimei University, Xiamen, China; ^2^National Research and Development Center for Eel Processing Technology, Key Laboratory of Eel Aquaculture and Processing of Fujian Province, Fujian Provincial Engineering Research Center for Eel Processing Enterprise, Changle Juquan Food Co. Ltd., Fuzhou, China

**Keywords:** *Edwardsiella tarda*, aptamer, SYBR green I real-time quantitative PCR, minimum detection limit, aptamer-qPCR

## Abstract

The *Edwardsiella tarda* bacterium can infect a wide variety of fish species and is a common pathogen in aquaculture. Rapid and accurate detection of the pathogen is the premise and basis for its disease prevention and control. In this study, an aptamer with high affinity and specificity was used to bind *E. tarda.* The aptamers that bound to the pathogen were then separated and used as the templates for SYBR Green I real-time quantitative polymerase chain reaction (qPCR) amplification. The Ct values obtained by qPCR can be used to quantitatively analyze the concentration of *E. tarda*, thereby establishing an aptamer-qPCR method for the quantitative detection of the pathogen with good specificity. Results showed that the Ct value of *E. tarda* was significantly lower than that of non-target bacteria (*Pseudomonas plecoglossicida, Pseudomonas aeruginosa, Escherichia coli, Vibrio anguillarum, Vibrio alginolyticus, Vibrio harveyi* and *Aeromonas hydrophila*) (*p* < 0.01). It had a good quantitative detection effect and showed good linearity in the range of 1–10^9^ CFU/mL. This method also had high sensitivity and stability, with minimum detection limit reaching 1 CFU/mL. This method was used to detect *E. tarda* in spiked water and tissue samples, proving its applicability for the detection of *E. tarda* in aquatic products, foods, and in the aquatic environment.

## 1 Introduction

*Edwardsiella tarda* of the Enterobacteriaceae family is a non-spore forming Gram-negative short bacillus with flagella, capable of surviving under anaerobic conditions (1). The pathogen can infect a variety of farmed fishes such as flatfish, turbot, and grass carp (2–6), posing a serious threat to the aquaculture industry. *E. tarda* is a zoonotic pathogen, causing low fever, gastroenteritis, liver abscess, meningitis, sepsis, and even death in humans that come into contact with water or food contaminated by *E. tarda* (7). Therefore, it is necessary to prevent and control this disease. Rapid and accurate detection of the bacterium is the premise and basis for its prevention and control.

Aptamers are oligonucleotide molecules screened by SELEX (Systematic Evolution of Ligands by Exponential enrichment) technology. Aptamers have many advantages such as high affinity, specificity, easy synthesis and modification, no immunogenicity, and a wide target range (8, 9), and have been widely used in the detection of various small targets such as metal ions, small molecules, proteins, bacteria, and cells (10–14). Therefore, aptamers are expected to efficiently detect *E. tarda*.

In this paper, an aptamer with good affinity and specificity for *E. tarda* obtained in a previous study (15), was used to identify and bind *E. tarda* in bullfrog tissue, and ultimately establish an aptamer-qPCR quantitative detection method for this pathogen. The specificity and sensitivity of this method was also assessed. This research is of great significance for the development of aptamers and disease control of *E. tarda*.

## 2 Materials and methods

### 2.1 Materials

#### 2.1.1 Aptamer and primers

Aptamer M1 used in the present paper was obtained from our previous study (15), which has high affinity and specificity for *E. tarda*. The sequence of aptamer M1 was 5’-TCAGTCGCTTCGCCGTCTCCTTCCGATCACTGTTGACCTAGTGGGGATGCGTCAGGGATAAGGGTGCACAAGAGGGA GACCCCAGAGGG-3′, and the sequences of primer P1 and P2 were 5’-TCAGTCGCTTCGCCGTCTCCTTC-3′ and 5’-CCCTCTGGGGTCTCCCTCTTGTGC-3′, respectively. The aptamer was provided by Beijing Tsingke Biotech Co., Ltd. and the primers were synthesized by Shenggong Biotechnology (Shanghai) Co., Ltd. Both the aptamer and the primers were prepared to a concentration of 10 μmol/L using a 2 × binding buffer.

#### 2.1.2 Buffer solution

The composition of the 20 × binding buffer solution was 1 × 10^6^ μM NaCl, 5 × 10^4^ μM KCl, 5 × 10^5^ μM Tris–HCl, and 1 × 10^4^ μM MgCl_2_, with a pH of 7.4. For use, this was diluted with sterile ultrapure water to form a 2 × binding buffer and a 1 × binding buffer. The PCR premix buffer system used for the qPCR (containing SYBR Green I dye) was purchased from Accurate Biotechnology (HuNan) Co., Ltd. (Changsha, China).

#### 2.1.3 Bacteria and culture media

Eight bacteria were used in this study, namely *E. tarda, Escherichia coli*, *Pseudomonas aeruginosa, Pseudomonas plecoglossicida, Vibrio anguillarum, Vibrio alginolyticus, Vibrio harveyi* and *Aeromonas hydrophila* were provided by the Pathogenic Microbiology Laboratory of Jimei University. *E. coli, P. aeruginosa* and *P. plecoglossicida* were cultured in a Luria-Bertani (LB) medium, and all other bacteria were cultured in a Tryptone Soy Broth (TSB) medium. The eight bacteria were all cultured in a 100 r/min shaker at 28°Cfor 12 h.

### 2.2 Method

#### 2.2.1 Linear relationship between aptamer concentration and Ct value

The aptamers were diluted with the 2 × binding buffer solution to 1, 10, 20, 30, 40, 50, 70, 90, and 100 pmol/L, respectively. Each of these solutions was denatured by heating at 95°C for 5 min, followed by an ice bath for 10 min, and then used as templates for the qPCR. The 20 μL qPCR reaction system consisted of 1 μL of the template, 10 μL of 2 × PCR premix (including SYBR Green I dye), 10 μmol/L of 1 μL primer P1, 10 μmol/L of 1 μL primer P2, and 7 μL ddH_2_O. The amplification parameters of the qPCR were as follows: pre-denaturation at 95°C for 3 min, followed by 40 cycles of denaturation at 95°C for 3 s and extension at 60°C for 30 s. A linear fitting was then performed with aptamer concentration on the x-axis and Ct value on the y-axis to obtain the linear curve and the equation of the Ct value-aptamer concentration. Using this equation the Ct value can be converted to the corresponding aptamer concentration.

#### 2.2.2 Specificity of the aptamer-qPCR method

Samples of the eight types of bacteria (*E. tarda, E. coli*, *P. aeruginosa, P. plecoglossicida, V. anguillarum, V. alginolyticus, V. harveyi* and *A. hydrophila*) were each diluted to 10^8^ CFU/mL with the 2 × binding buffer. The 200 nmol/L aptamers were first denatured by heating at 95°C for 5 min, followed by a 10-min ice bath (all aptamers used in subsequent experiments were treated this way). In the experimental group, 100 μL of each 10^8^ CFU/mL suspension of the five bacteria was mixed with 100 μL of 200 nmol/L treated aptamers. In the control group containing bacteria, 100 μL of the 2 × binding buffer was used to replace the aptamer to mix with 100 μL of the bacterial suspension at 10^8^ CFU/mL. In the control group containing no bacteria, 100 μL of 2 × binding buffer was used instead of the bacterial suspension to mix with 100 μL of 200 nmol/L treated aptamer. Both the experimental and control groups were incubated on a shaker at 25°C and 200 r/min (revolutions per minute) for 1 h. They were then centrifuged at 1200 × g for 5 min to remove the supernatant. The bacterial pellet was washed four times with the 1 × binding buffer to wash away the unbound aptamers. After centrifugation, the supernatant was discarded. The bacterial suspension was obtained by resuspending the bacterial pellet with 100 μL of the 1 × binding buffer. The bacterial suspension was then heated at 95°C for 5 min, cooled, and centrifuged at 7500 × g for 10 min. The supernatant was diluted 1,000 times and stored at 4°C.

The diluted supernatant was used as template for the qPCR amplification. The qPCR system and parameters were the same as those in section 2.2.1. After completing the qPCR, the corresponding Ct values were recorded. Then, based on the linear equation obtained in section 2.2.1, the concentration of aptamers in the undiluted supernatant (i.e., the concentration of the aptamer bound to the bacteria) was calculated.

#### 2.2.3 Quantitative detection of *Edwardsiella tarda* by the aptamer-qPCR method

*Edwardsiella tarda* was diluted to 1, 10, 10^2^, 10^3^, 10^4^, 10^5^, 10^6^, 10^7^, 10^8^, 10^9^ CFU/mL, respectively, using the 2 × binding buffer. The experimental and control groups were prepared according to method in section 2.2.2. The bacterial suspension of 10^4^ CFU/mL was selected for the control group containing bacteria.

Following the method in section 2.2.2 the experimental group and the two control groups were incubated, centrifugated, washed, the bacterial pellet was resuspended, and the solution was heated and centrifugated again. Finally, the obtained supernatant was diluted 1,000 times and stored at 4°C.

The diluted supernatant was used as the template for the qPCR, where the system and parameters were same as those in section 2.2.1. A standard curve was made using the logarithm of the bacterial concentrations and the Ct values of the qPCR to assess the quantitative detection effect of this aptamer-qPCR method.

#### 2.2.4 Effects of aptamer concentration and binding time

The aptamers were diluted with the 2 × binding buffer to 20, 30, 40, 50, 80, 100, 150, 200, 250, and 300 nmol/L, respectively. Then, 100 μL of each of these was mixed with 100 μL of 10^8^ CFU/mL *E. tarda* solution and incubated in a shaker at 25°C and 200 r/min for 1 h. Subsequently, each of these was centrifuged at 1200 × g for 5 min, the supernatant was discarded, and the bacterial pellet was washed 4 times with the 1 × binding buffer. After centrifugation, the supernatant was discarded, and the bacterial pellet was resuspended with 100 μL of the 1 × binding buffer to obtain the bacterial suspension. The bacterial suspension was heated at 95°C for 5 min, then cooled and centrifuged at 7500 × g for 10 min. The supernatant contained the aptamer bound to the bacteria which was diluted 1,000 times and used as the template for the qPCR. The qPCR system and parameters were the same as those in section 2.2.1. The concentration of the binding aptamer was determined based on the Ct values obtained from the qPCR and the linear equation of the Ct-aptamer concentration acquired in 2.2.1. This was then plotted with the concentration of the binding aptamer on the y-axis and the concentration of the added aptamer on the x-axis to assess the influence of aptamer concentration on its binding properties and to determine the appropriate aptamer addition concentration.

Twenty-seven centrifuge tubes were used and in each 100 μL of 10^8^ CFU/mL *E. tarda* and 100 μL of 200 nmol/L aptamer were added. The tubes were then incubated in a shaker at 25°C and 200 r/min and randomly removed at 10, 20, 30, 40, 50, 60, 70, 90, and 100 min, respectively. They were centrifuged at 1200 × g for 5 min, the supernatants were removed, and the bacterial pellets were washed 4 times and resuspended with 100 μL of 1 × binding buffer to obtain the bacterial suspension. The bacterial suspension was then heated at 95°C for 5 min, cooled, and centrifuged at 7500 × g for 10 min. The obtained supernatant contained the aptamers bound to the bacteria. The supernatant was diluted 1,000 times and used as the template for the qPCR. The qPCR parameters and system were the same as those in 2.2.1 and were used to determine the appropriate binding time for the aptamer and the bacteria.

#### 2.2.5 Application detection

To study the detection effects of the aptamer-qPCR method on *E. tarda* in water and biological tissue samples, spiked recovery experiments were conducted using fresh water, seawater of different salinities, and soaking solutions of different bullfrog tissues. The sample preparation was a slightly modified version of the method reported by Huang et al. (16). Freshwater samples were prepared using ultrapure water, while seawater samples were prepared using seawater crystals purchased from Jinan Yande Biotechnology Co., Ltd. The tissue samples were made by cutting the organ tissues from bullfrogs into small 0.5 g pieces which were then soaked in 5 mL of the 2 × binding buffer for 1 h, followed by centrifugation after which the supernatant obtained was the tissue suspension. Subsequently, 2 mL of bacterial suspensions of 2 × 10^3^ and 2 × 10^4^ CFU/mL were mixed with 2 mL of the freshwater, seawater, and tissue samples to prepare the corresponding spiked samples, resulting in concentrations of 10^3^ and 10^4^ CFU/mL of *E. tarda* in these spiked samples. After this, 300 μL of the spiked sample was mixed with 100 μL of 100 nmol/L aptamer and incubated at room temperature at 200 r/min for 1 h. The mixture was then centrifuged at 1200 × g for 5 min, the supernatant was discarded, and the bacterial pellet was washed 4 times with the 1 × binding buffer. Finally, 100 μL of the 1 × binding buffer was added to prepare the bacterial suspension, which was heated at 95°C for 5 min, cooled, and centrifuged at 7500 × g for 10 min. The supernatant contained the aptamer bound to the bacteria and was then diluted 1,000 times and used as the template for the qPCR. The qPCR was conducted according to the system and parameters of section 2.2.1, and the Ct value of each sample was obtained. The standard curve using the Ct and the logarithm of bacterial concentration was made following the method in section 2.2.2, and the bacterial concentration of each sample was calculated based on this curve. The recovery rate of the bacteria was calculated as follows: recovered volume/added volume ×100%.

#### 2.2.6 Statistical analysis

All experiments were performed using three replicates, and *t*-tests were performed to analyze inter-group differences in the experimental data, with *p* < 0.05 indicating significant differences, and *p* < 0.01 indicating extremely significant differences.

## 3 Results

### 3.1 Detection principle of the aptamer-qPCR method

**Figure 1:**
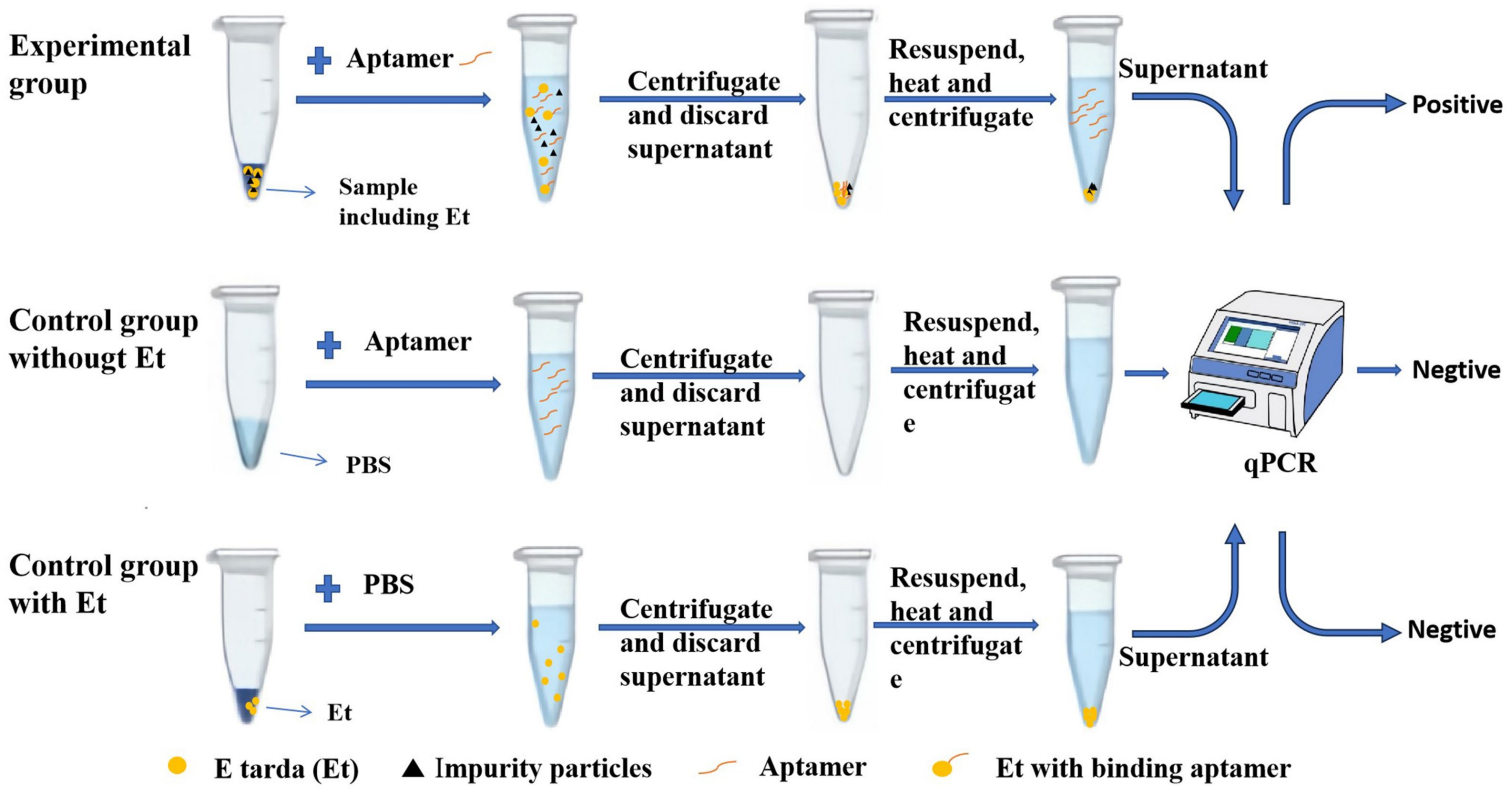
Schematic diagram of the qPCR method for the detection of *Edwardsiella tarda* based on an aptamer.

As shown in Figure 1, *E. tarda* (Et) was the target bacterium for detection, and the aptamer was the recognition molecule that can specifically bind to this pathogen. In the experimental group containing *E. tarda*, the aptamer bound to the bacterium. Centrifugation yielded bacterial precipitates containing the bacterium and its bound aptamer, as well as some precipitated impurity particles. Subsequently, the precipitate was resuspended and heated, causing the bacterium to rupture, and releasing the bound aptamer into the solution. After another centrifugation the precipitate consisted of the bacterial fragments and impurity particles, while the supernatant contained the bound aptamers. Using the aptamers in the supernatant as templates for the qPCR, the corresponding Ct value was obtained. The higher the concentration of *E. tarda*, the greater the number of aptamers bound to the bacterium, and the lower the Ct value. A quantitative assessment (see section 3.2 below) indicated a linear relationship between the logarithm of the concentration of *E. tarda* and its Ct value. Therefore, the concentration of *E. tarda* can be quantitatively analyzed by measuring the Ct value.

Two control groups were set up to exclude the contamination of *E. tarda* and its aptamer in the detection system, respectively. The control group without *E. tarda* was used to identify whether this target bacterium was mixed into the detection system. Although this control group contained the aptamer, since there was no *E. tarda* in the detection system, the normal qPCR test should be negative without a Ct value. However, if the system is contaminated with *E. tarda*, the qPCR test would show a false positive with a Ct value. The second control group containing *E. tarda* was used to assess whether the *E. tarda* sample used to make the standard curve was contaminated by the aptamer. Normally, this should not contain any aptamer, so the normal qPCR test should also be negative with no Ct value. If the *E. tarda* samples are contaminated by the aptamers, the qPCR test will show false positive with Ct values. Only when both the control groups are negative can we ensure the reliability of the detection of *E. tarda* in the experimental group.

### 3.2 Specificity and quantitative effect of the aptamer-qPCR method

**Figure 2:**
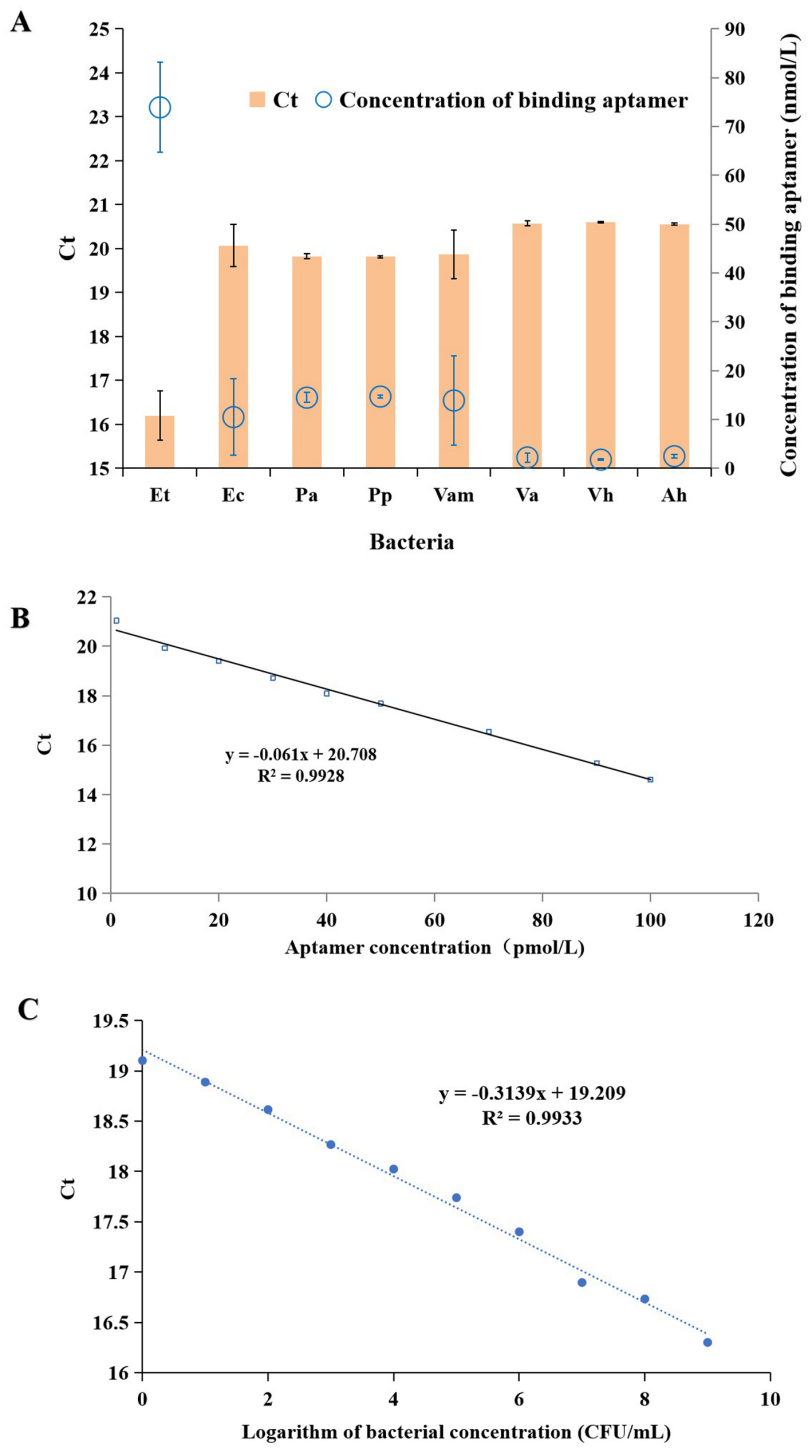
Detection of *Edwardsiella tarda* (Et), *Escherichia coli* (Ec), *Pseudomonas aeruginosa* (Pa), *Pseudomonas plecoglossicida* (Pp), *Vibrio anguillarum* (Vam), *Vibrio alginolyticus* (Va), *Vibrio harveyi* (Vh), and *Aeromonas hydrophila* (Ah) by the aptamer-qPCR method **(A)**, the linear curve of Ct and aptamer concentration **(B)**, and standard curve for the quantitative detection of *E. tarda* using aptamer-qPCR **(C)**.

As shown in Figure 2A, *E. tarda* and the seven other bacteria (*E. coli, P. aeruginosa, P. plecoglossicida, V. anguillarum, V. alginolyticus, V. harveyi* and *A. hydrophila*) were detected by the aptamer-qPCR method. The results indicate that there was no amplification signal in both control groups, suggesting that the experimental process was free from contamination. The Ct value of *E. tarda* was significantly lower than those of the other seven bacteria (*p* < 0.01). Using the linear equation of the Ct-aptamer concentration (Figure 2B), the Ct values corresponding to the bacteria can be converted into the concentrations of binding aptamers. The results showed that the concentration of binding aptamers for *E. tarda* was also significantly higher than those of the other seven bacteria (*p* < 0.01) (Figure 2A), indicating that this method has good detection specificity for *E. tarda*.

Nine concentrations (1, 10, 10^1^, 10^2^, 10^3^, 10^4^, 10^5^, 10^6^, 10^7^, 10^8^, 10^9^ CFU/mL) of *E. tarda* were detected by the aptamer-qPCR method, and the standard curves obtained from bacterial concentrations and Ct values are shown in Figure 2C. Here, the logarithm of bacterial concentration (CFU/mL) has a good linear relationship with the Ct values, with a linear fit coefficient R^2^ of 0.9933. The corresponding fitting equation is Ct = −0.3139X + 19.209, indicating that this detection method has a good linearity within the range of 1 to 10^9^ CFU/mL. Therefore, the aptamer-qPCR method can be used for the quantitative detection of *E. tarda*, with a minimum detection limit (LOD) of 1 CFU/mL.

### 3.3 Effects of aptamer concentration and its binding time on detection capabilities

**Figure 3:**
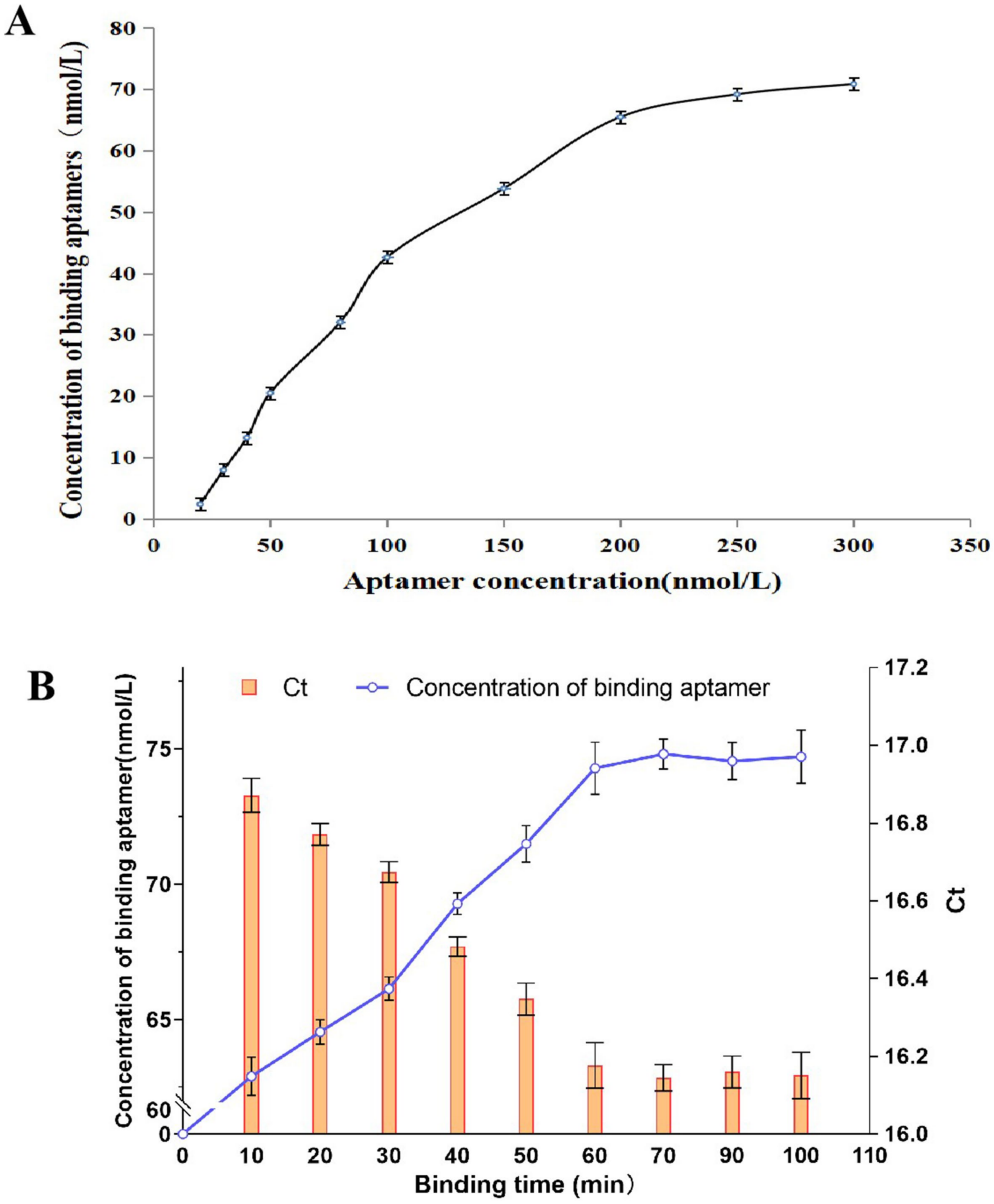
The effects of aptamer concentration **(A)** and the binding time **(B)** on the binding of aptamer to *E. tarda*.

As shown in Figure 3A, the concentration of the aptamer bound to *E. tarda* increased with an increase in aptamer concentration, with a decreasing slope. When 200 nmol/L was exceeded, the curve gradually flattened out, indicating that further increasing the aptamer addition concentration has a reduced impact on the binding properties of the aptamer to the bacterium. This shows that the *E. tarda* in the system has been saturated by the aptamers, therefore, we chose 200 nmol/L of aptamer to mix with *E. tarda* in equal volumes.

Figure 3B shows that as the binding time increased, the concentration of the binding aptamer on the bacterium gradually increased, and the corresponding Ct values gradually decreased. After 60 min, the concentration of the binding aptamer no longer significantly increased (*p* > 0.05), and the Ct values no longer significantly decreased (*p* > 0.05), indicating that the aptamer has reached stability. Therefore, we chose 60 min as the binding time between the aptamer and the bacteria in the detection.

### 3.4 Application detection

The bacterium *E. tarda* in the spiked water and tissue samples was detected by the aptamer-qPCR method, and the results are shown in Table 1. The relative standard deviation was 0.001 to 1.055%, and the recovery rate was 60.107 to 172.38%. Salinity had no significant influence on the test results (*p* > 0.05). The detection data of water samples showed less fluctuation, while those of tissue samples showed more fluctuation. Generally, the relative standard deviation should be less than 12% (16, 17). According to the requirements for microbial detection in the 2020 edition of the Pharmacopeia of the People’s Republic of China, the recovery rate should be between 50 and 200% (18). The detection of both the spiked water and tissue samples met these requirements, indicating that this aptamer-qPCR method is accurate and reliable, and can be used for the detection of *E. tarda* in water and biological tissue.

**Table 1:** Application test of spiked water and tissue samples.

Samples	Bacterial concentration (CFU/mL)	Average Ct	Standard deviation	Relative standard deviation %	Recovery rate %
Freshwater samples	10^3^	18.275	0.078	0.425	94.740
	10^4^	17.981	0.140	0.780	82.062
Seawater samples 1 (2% salinity)	10^3^	18.307	0.038	0.002	74.537
	10^4^	17.920	0.016	0.001	127.101
Seawater samples 2 (2.5% salinity)	10^3^	18.284	0.080	0.438	88.362
	10^4^	17.927	0.081	0.454	121.368
Seawater samples 3 (3% salinity)	10^3^	18.301	0.030	0.163	73.827
	10^4^	17.925	0.003	0.346	122.861
Seawater samples 4 (3.5% salinity)	10^3^	18.337	0.129	0.007	60.107
	10^4^	17.928	0.077	0.004	120.151
Seawater samples 5 (4% salinity)	10^3^	18.303	0.081	0.004	77.133
	10^4^	17.936	0.014	0.001	113.580
Epidermis	10^3^	18.307	0.100	0.547	74.918
	10^4^	17.914	0.058	0.325	133.675
Muscles	10^3^	18.29	0.008	0.770	84.661
	10^4^	17.915	0.077	0.430	132.860
Heart	10^3^	18.285	0.007	0.708	87.824
	10^4^	17.923	0.077	0.429	124.676
Liver	10^3^	18.246	0.193	1.055	118.048
	10^4^	17.884	0.052	0.288	166.066
Spleen	10^3^	18.247	0.083	0.455	116.621
	10^4^	17.879	0.090	0.005	172.380

## 4 Discussion

Currently, the most common methods for detecting *E. tarda* include traditional microbial cultures, immunology, and molecular biology based on specific genes. Most of the commercially available reagent kits are microbial culture methods, which require bacterial cultivation, have a long detection time, low sensitivity, and are difficult to conduct quantitative detection (19, 20). Molecular biology methods mainly include the 16S rDNA and PCR methods based on specific genes (7, 21–24). The 16S rDNA method is generally used for the qualitative identification of bacteria and is less frequently used for quantitative detection (7, 21), and other PCR methods (multiplex PCR and qPCR, and digital PCR) mainly target virulence-related genes such as esrB, gadB, type III secretion system (T3SS), and gyrB (single-copy DNA gyrase subunit) in *E. tarda*. Most PCR methods are qualitative or semi-quantitative, whereas quantitative techniques, including digital PCR and qPCR, typically have detection limits ranging from 0.56 copies/μL (approximately 560 CFU/mL) to 10^2^ copies/μL (around 10^5^ CFU/mL) (7, 24). Overall, molecular biology requires the extraction of bacterial DNA, the design of primers targeting particular genes, and the high specificity and copy number of the selected genes. In immunology for *E. tarda* the minimum LOD of the ELISA method established using monoclonal antibodies was 1 × 10^4^ CFU/mL (25), and the minimum LOD of colloidal gold immunochromatography method was 5 × 10^6^ CFU/mL (26). Although the antibody method is easy to use, its sensitivity is too low. Moreover, the development and preparation costs of monoclonal antibodies are relatively high, limiting their practical application. The aptamer-qPCR method established in this article can quantitatively detect *E. tarda* with a minimum LOD of 1 CFU/mL, currently ranking among the top detection methods for *E. tarda* in water and tissue samples.

Aptamers have been used in the detection of many kinds of bacteria, and various aptamer-based detection technologies including fluorescence, gold nanoparticles, and electrochemical sensing, have been developed (27, 28). Although there are various aptamers, target bacteria, and detection techniques, the minimum LOD for bacteria is generally within the range of 1–10^6^ CFU/mL. In contrast, our aptamer-qPCR method has a quantification range of 1–10^9^ CFU/mL and a minimum LOD as low as 1 CFU/mL, currently ranking among the top in the aptamer-based bacterial detection methods.

In addition, we also found that the recovery rate of liver and spleen tissues fluctuated more compared to all other tissue and water samples. This was probably due to the higher number of endogenous impurities, such as proteins and nucleic acids, in these samples which may non-specifically bind to aptamers, causing abnormal fluctuations in the Ct values of the qPCR. The interference of impurities in the tissue samples within the detection results has also been reported in some other studies (28–30). Nevertheless, the results indicate that the recovery rate of the spiked test was still within the allowable error range (50–200%), suggesting that the influence of this interference on the detection of *E. tarda* is low.

## 5 Conclusion

In the present study, an aptamer-qPCR method for the detection of *E. tarda* was successfully developed by combining aptamers with high specificity and qPCR with high sensitivity. The detection method had high specificity for *E. tarda* and could quantitatively detect the pathogen in the range of 1–10^9^ CFU/mL with a minimum LOD of 1 CFU/mL. The feasibility of this method was further verified by the application of spiked water and tissue samples, providing evidence that this method can be used to detect *E. tarda* in the aquatic environment and in aquatic products.

## Data Availability

The raw data supporting the conclusions of this article will be made available by the authors, without undue reservation.
